# Adenoviral gene transfer of bioactive TGFβ1 to the rodent eye as a novel model for anterior subcapsular cataract

**Published:** 2007-03-27

**Authors:** Jennifer V. Robertson, Zahra Nathu, Anas Najjar, Dhruva Dwivedi, Jack Gauldie, Judith A. West-Mays

**Affiliations:** 1 Department of Pathology and Molecular Medicine, McMaster University, Hamilton, ON, Canada; 2 Centre for Gene Therapeutics, McMaster University, Hamilton, ON, Canada

## Abstract

**Purpose:**

To produce a gene-transfer model of rodent anterior subcapsular cataracts (ASC) using a replication-deficient, adenoviral vector containing active TGFβ1. Establishment of this model will be important for further investigations of TGFβ-induced signaling cascades in ASC.

**Methods:**

Adenovirus containing the transgene for active TGFβ1 (AdTGFβ1), β-galactosidase (AdLacZ), green fluorescent protein (AdGFP) or no transgene (AdDL) was injected into the anterior chamber of C57Bl/6, Smad3 WT and Smad3 KO mice. Four and 21 days after injection, animals were enucleated and eyes were processed and examined by routine histology. Immunolocalization of markers indicative of epithelial to mesenchymal transition (EMT), fibrosis, proliferation and apoptosis was also carried out.

**Results:**

By day 4, treatment with AdLacZ demonstrated transgene expression in multiple structures of the anterior chamber including the lens epithelium. In contrast to AdDL, treatment with AdTGFβ1 produced αSMA-positive subcapsular plaques in all three groups of mice, which shared features reminiscent of human ASC. At day 21, plaques remained αSMA-positive and extensive extracellular matrix deposition was observed. The AdTGFβ1 model was further employed in Smad3 deficient mice and this resulted in the development of small ASC.

**Conclusions:**

Gene transfer of active TGFβ1 using an adenoviral vector produced cataractous plaques four days postinjection, which were found to develop independent of functional Smad3.

## Introduction

The family of transforming growth factor beta (TGFβ) molecules is a group of secreted polypeptides with functions in cellular proliferation, differentiation and migration as well as extracellular matrix metabolism [1]. Considerable evidence has shown that TGFβ1 and 2 are potent profibrotic molecules that have been implicated in numerous fibrotic diseases including diabetic nephropathy, liver and pulmonary fibrosis, rheumatoid arthritis, and more recently, ocular fibrotic diseases [2].

Ocular pathologies associated with aberrant levels of active TGFβ include cataracts, proliferative vitreoretinopathy (PVR), as well as glaucoma [3-6]. The active form of TGFβ has been detected in the ocular media from patients suffering with cataracts [7,8] and in patients undergoing intraocular lens implantation [9]. Moreover, patients treated with TGFβ2 to treat retinal macular holes all developed cataracts after treatment [10]. Two specific types of cataracts are associated with elevated levels of activated TGFβ, including anterior subcapsular cataracts (ASC) and posterior capsular opacification (PCO), also known as secondary cataract.

In ASC, focal opacities develop beneath the lens capsule in the anterior region of the lens. These opacities or plaques have been shown to be derived from an aberrant proliferation of lens epithelial cells (LECs). A proportion of these cells also undergo a transition into myofibroblasts, which express alpha-smooth muscle actin (αSMA), through a mechanism known as epithelial to mesenchymal transformation (EMT) [11]. As the cataracts further develop, the plaque cells secrete extracellular matrix (ECM) components not found in the normal lens, such as collagen type I and IV, contributing to the loss of lens transparency. PCO remains the major complication of modern cataract surgery with intraocular lens implantation [12]. In PCO, the LECs which remain within the capsule after cataract surgery are triggered to proliferate and migrate to the posterior lens capsule [13] and like ASC, some of these cells undergo a transition into myofibroblasts that secrete aberrant ECM.

Multiple in vitro and in vivo rodent models have been developed for studying the mechanism(s) of TGFβ-induced ASC. In vitro models include excised rat lenses, lens epithelial explants and cell cultures that when exposed to TGFβ undergo morphological changes similar to that seen in human ASC including LEC transformation and inappropriate ECM deposition [14-18]. Ectopic expression of active TGFβ1 in the lens of transgenic mice under the transcriptional control of the αA-crystallin [19] or βB-crystallin [20] promoter also results in ASC plaques that resemble those observed in humans. More recently, two in vivo injury models for ASC have been developed in mice including direct injury to the lens with a hypodermic needle, as well as an alkali burn to the ocular surface [21,22]. In both of these models LECs are stimulated to undergo EMT through activation of TGFβ2. While the TGFβ signaling intermediate, Smad3, was shown to be required for the EMT observed in the lens injury model, in the alkali burn model both EMT and ASC were shown to occur in the absence of Smad3, albeit the effect was attenuated. Similarly, our lab has shown that when the lens specific-TGFβ1 transgenic mice were bred onto the Smad3 knockout mouse background, small ASC formed [23]. Thus, multiple models have shown that alternative TGFβ signaling pathways participate in the development of ASC.

Our group has shown that transient overexpression of bioactive TGFβ1 and β3 using adenoviral vectors results in accumulation of parenchymal extracellular matrix and EMT of epithelial cells, as demonstrated by increased expression of αSMA in both the lung [24] and in the peritoneum [25]. Moreover, we have shown that adenoviral transfer of TGFβ1 produces transgene detectable by RNAse protection assay, ELISA and a functional reporter assay, confirming transgene activity [24,26,27]. We posited whether adenoviral transfer of TGFβ1 to the anterior segment of the rodent eye could be used to induce EMT of LECs and create a novel model of ASC that would not require injury or developmental expression of a TGFβ transgene. Using adenoviral-LacZ (AdLacZ) we have shown successful adenoviral transduction of a number of tissues within the anterior segment of the eye, including the lens epithelium. We further demonstrate that transfer of adenoviral-(active) TGFβ1 (AdTGFβ1) to cells of the rodent anterior segment resulted in the development of ASC plaques, within 4 days of injection, which continued to progress into large fibrotic plaques by day 21. Finally, we employed this new model to show that delivery of AdTGFβ1 to the anterior chamber of genetically modified mice deficient in Smad3, results in the formation of ASC plaques, confirming our earlier findings that Smad3-independent signaling mechanisms participate in the EMT of LECs in ASC formation.

## Methods

### Recombinant adenoviruses

Full-length porcine TGFβ1 cDNA was mutated at serines 223 and 225 (TGFβ1^223/225^) to render the expressed protein product constitutively and biologically active [28]. This mutated cDNA was used to construct a recombinant, replication-deficient type-5 adenovirus. The E1 region was replaced by the human cytomegalovirus promoter, driving expression of TGFβ1^223/225^ followed by the SV40 polyadenylation signal [24]. The resulting replication-deficient virus (AdTGFβ1) was amplified and purified by cesium chloride (CsCl) gradient centrifugation and concentrated using a Sephadex PD-10 chromatography column, and finally plaque-titered on 293 cells. The control vectors, AdDL, with no insert in the deleted E1 region, or adenoviral vector expressing the β-galactosidase gene (AdLacZ), coding for β-galactosidase, or adenoviral vector expressing GFP (AdGFP), were produced by similar methods [24,29].

### Animal treatment

All animals were treated in accordance with the guidelines of the Canadian Council on Animal Care and according to the ARVO Statement for the Use of Animals in Ophthalmic and Vision Research. Female, 6- to 8-week-old C57BL/6 and FVB/n mice were purchased from Charles River Laboratories (Montreal, PQ, Canada). Smad3 knockout mice were generated by the removal of exon 8 of the Smad3 gene in mice of background 129SV/EV x C57BL/6 [30]. Smad3 heterozygous mice were bred under special pathogen-free conditions. The genotypes of both wildtype (WT) and Smad3 knockout (Smad3KO) mice were determined by PCR analysis on tail DNA obtained from 3-wk-old animals. Tail biopsies were digested overnight at 65 °C in buffer containing 0.1 M TrisHCl (pH 8.5), 5 mM EDTA (pH 8), 0.2% SDS, 200 mM NaCl and 0.2 mg/ml Proteinase K (Bioshop Canada, Burlington, ON). Digested samples were spun down and supernatants were mixed with 100% ethanol to precipitate genomic DNA, which was then diluted in 100 μl of sterile distilled water. Primer sequences were as follows: S3P1 5'-CCA CTT CAT TGC CAT ATG CCC TG-3'; S3P2 5'-CCC GAA CAG TTG GAT TCA CAC A-3'; S3P3 5'-CCA GAC TGC CTT GGG AAA AGC-3'. The wild type allele was generated using primers S3P1 and S3P2 giving a 400 bp fragment; the knockout allele was generated using primers S3P1 and S3P3 giving a 250 bp fragment. PCR product was run on a 2% TAE agarose gel and visualized by ethidium bromide staining under UV light. All experiments were performed with littermates to rule out any background effects. All animals were housed under specific pathogen free conditions and rodent laboratory food and water were provided ad libitum. All animal procedures were performed under inhalation anesthesia with isofluorane (MTC Pharmaceuticals, Cambridge, ON, Canada). AdTGFβ1 or AdDL or AdLacZ (5x10^8^ pfu) were administered in a volume of 5 μl phosphate-buffered saline (PBS). Briefly, mice were anesthetized with isofluorane and placed under a dissecting microscope, in order to visualize general eye structures. A volume of no more than 5 μl of virus solution was injected into the anterior chamber using a 33 gauge needle affixed to a 10 μl Hamilton syringe. Eyes were covered with Lacri-lube® after injection and animals were allowed to recover before returning to their cages. Animals were sacrificed and enucleated 4 or 21 days after injection.

### Histology

After fixation in 10% buffered formalin for 48 h, tissues were embedded in paraffin by routine methods. Three-micrometer thick midsagittal sections were cut and stained with hematoxylin and eosin to visualize general tissue architecture. Additional sections were stained with Masson's trichrome and periodic acid Schiff (PAS) to demonstrate ECM components. Immunohistochemistry using an antibody against α-smooth muscle actin (αSMA, clone 1A4; DakoCytomation, Mississauga, ON) was used to localize contractile elements. The brown color reaction was performed with the Animal Research Kit (DakoCytomation, Mississauga, ON). Studies to fluorescently colocalize αSMA employed a monoclonal antibody conjugated to FITC (clone 1A4; Sigma-Aldrich, Oakville, ON). In addition, sections were stained with polyclonal antibodies to β- and γ-crystallin (kindly provided by Dr. J.S. Zigler, National Eye Institute, Bethesda, MD), collagen IV (Cedarlane Laboratories, Hornby, ON), PCNA, and TGFβ1 (Santa Cruz Biotechnologies, Santa Cruz, CA). Secondary antibodies included goat anti-rabbit rhodamine and goat anti-rabbit FITC (Bioshop Canada, Burlington, ON). TUNEL staining was carried out using the ApopTag® Fluoescein In Situ Apoptosis Detection Kit according to the manufacturer's instructions (Millipore, Temecula, CA).

### LacZ staining and GFP visualization

All reagents were purchased from Bioshop Canada (Burlington, ON). Directly after enucleation, eyes were placed whole in fixative buffer containing 0.2% glutaraldehyde, 5 mM EGTA pH 7.3, 2 mM MgCl_2_ in 0.1 M sodium phosphate buffer pH 7.3 for 30 min. They were then washed three times of 5 min each with wash buffer containing 2 mM MgCL_2_, 0.01% deoxycholate, 0.02% Nonidet P-40 in 0.1 M sodium phosphate buffer, pH 7.3. Staining was carried out overnight at 37 °C in staining buffer containing 2.0 ml of 25 mg/ml X-gal, 0.106 g potassium ferrocyanide and 0.082 g potassium ferricyanide in 50 ml of wash buffer. After staining, tissues were washed three times of 5 min each and placed in 10% neutral buffered formalin for an additional 24 h to ensure adequate fixation prior to sectioning. Three micrometer thick midsagittal sections were cut and counterstained with nuclear fast red using routine methods.

Frozen sections from eyes treated with AdGFP were kept at -20 °C until use. Sections were then allowed to come to room temperature, washed three times for 5 min with PBS and coverslipped with Vectashield® mounting medium with DAPI (Vector Laboratories, Burlinghame, CA).

## Results

### Anterior structures of the eye can be transduced with adenovirus four days post injection

**Figure 1:**
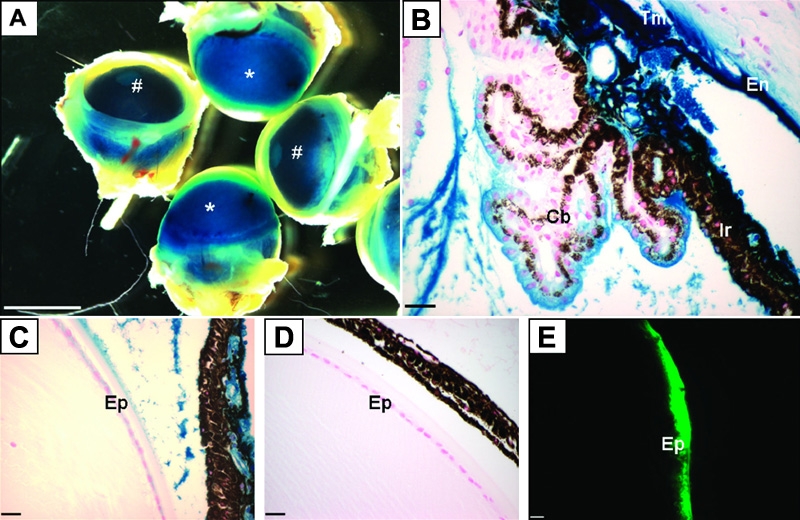
Expression of adenovirally transferred LacZ and GFP. Animals were injected with AdLacZ (**A**-**C**), AdDL (**D**), or AdGFP (**E**) intracamerally. Four days post injection eyes were removed and wholemount stained for LacZ activity (**A**) followed by paraffin sectioning (**B**,**C**,**D**) or frozen sectioning and visualized using a GFP filter (**E**). Blue corneal opacity (asterisks in **A**) indicates that cellular infection and production of transgene was successful. AdDL eyes remained clear (hash marks in **A**). **B** and **C** demonstrate transgene expression in the corneal endothelium (En), iris (Ir), ciliary body (Cb), trabecular meshwork (Tm), and lens epithelium (Ep). Transgene was absent in control vector treated eyes (**D**). Transgene expression is confirmed in the lens epithelium (Ep) using AdGFP (green). The scale bars in **A** = 1 mm, in **B**-**D** = 25 μm, and in **E** = 50 μm.

To determine the cell and tissue types in the anterior chamber of the eye that can be infected with adenovirus, we injected reporter constructs, AdLacZ (5x10^8^ pfu or 1x10^9^ pfu) or AdGFP (5x10^8^ pfu or 1x10^9^ pfu) intracamerally into mice of a C57BL/6 or FVB/n background, respectively. LacZ staining was then performed to localize transgene expression. Grossly, LacZ positive eyes could be distinguished from eyes injected with control virus containing no transgene (AdDL) simply by the bluish corneal opacity (Figure 1A) in contrast to the clear corneas of the AdDL-treated mice. Histological midsagittal sections of AdLacZ treated eyes revealed that after 4 days, LacZ staining was observed in multiple structures of the anterior chamber, including the corneal endothelium, iris, ciliary body and trabecular meshwork (Figure 1B). Transgene was also detectable in the lens epithelium (Figure 1C) in absence of capsular break, but typically at the higher dose (1x10^9^ pfu). Animals injected with the AdDL vector (Figure 1D) did not show any LacZ staining. Transduction of lens epithelial cells was also confirmed by expression of GFP (Figure 1E) after intracameral injection with AdGFP. In comparison to 4 days post-injection, 21 days after injection with AdLacZ, only faint LacZ staining was observed in the trabecular meshwork and corneal endothelium and none was observed in the lens (data not shown).

### Gene transfer of active TGFβ1 to the anterior chamber induces ASC formation

**Figure 2:**
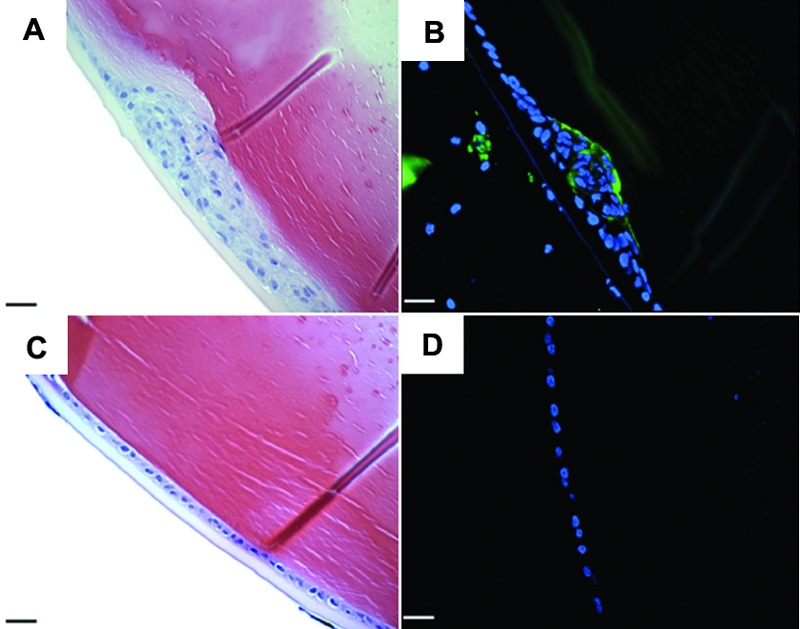
Effects of adenoviral gene transfer of active TGFβ1 after four days. Histological sections from C57 mice injected with AdTGFβ1 (**A** and **B**) or AdDL (**C** and **D**). Sections were stained with H&E (**A** and **C**) or subjected to immunostaining for αSMA (**B** and **D**). Focal multilayering occurs in animals treated with AdTGFβ and is associated with induction of αSMA (**A**, **B**) in contrast to AdDL treated eyes (**C**, **D**). The green stain is αSMA and the blue stain is DAPI. The scale bar is equal to 25 μm.

One eye of each mouse (6-8 weeks old; C57BL) was injected with AdTGFβ1 or AdDL. Four days post injection, 13 lenses out of 15 eyes treated with AdTGFβ1 (Figure 2A) showed distinct ASC plaques consisting of a focal multilayering of LECs beneath the intact anterior lens capsule. Each of the AdTGFβ1-treated eyes commonly exhibited one plaque, typically found in a central location of the anterior region of the lens. In comparison to those treated with AdTGFβ1, 2 lenses out of 15 eyes injected with AdDL exhibited subcapsular plaques in the absence of any lens rupture. However, these eyes also showed a moderate amount of inflammation, suggesting that the cataracts may have developed in response to excessive injury in the anterior segment of the eye. The remaining 13 eyes, showed a normal, lens epithelial monolayer (Figure 2C). Injection with either AdTGFβ1 or AdDL also produced a mild inflammatory response as evidenced by presence of a small number of neutrophils and macrophages both in the aqueous humor and within the corneal stroma. These effects were transient and absent by day 21. AdTGFβ1 treated eyes, however, demonstrated persistent thickening and increased cellularity of the corneal stroma, as well as a reduction in the number of corneal epithelial layers. Adhesions between the corneal endothelium and iris were also common. These non-lens features have also been reported in the TGFβ1-transgenic mouse models, whereby active TGFβ1 is overexpressed in lens fiber cells during embryogenesis [19,20]. These shared features are likely a result of TGFβ overexpression and not due to adenoviral infection. Importantly, AdDL animals did not show these features at any time-point examined and the contralateral/uninjected eyes remained normal during the entire course of the study (data not shown).

To determine whether plaques consisted of myofibroblasts, as previously reported in human ASC and animal models of ASC, sections of the day 4 AdTGFβ1- and AdDL-treated eyes were subjected to immunohistochemical staining for αSMA. In AdTGFβ1-treated eyes, distinct expression of αSMA was observed in a proportion of the cells in the plaques of the lenses which exhibited subcapsular plaques (Figure 2B). Expression was also observed in some of the adjacent monolayer of cells in the anterior lens region. However, the monolayer lens epithelium distal to the site in AdTGFβ1-treated eyes did not express αSMA. In comparison, AdDL treated eyes, showed no detectable expression of αSMA in the lens (Figure 2D).

**Figure 3:**
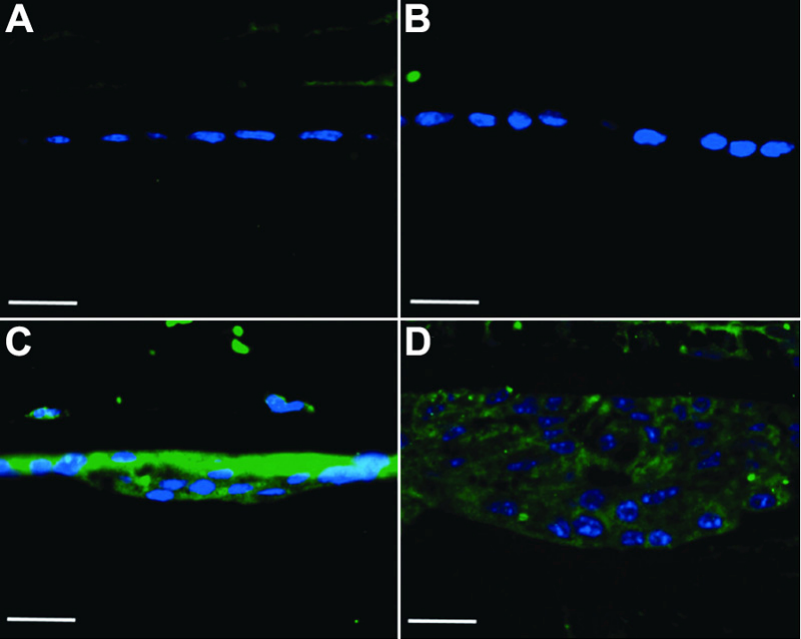
Immunolocalization of TGFβ1. Naïve (**A**), AdDL injected (**B**), and AdTGFβ1 injected eyes (**C** and **D**) were sectioned and subjected to TGFβ1 immunohistochemistry at 0 (**A**), 4 (**B** and **C**) and 21 (**D**) days after injection. Both naïve and AdDL eyes show no immunolocalization of TGFβ1 in the lens epithelium. Animals injected with AdTGFβ1 demonstrate prominent expression of TGFβ1 in the lens epithelium. The green stain is TGFβ1 and the blue stain is DAPI. The scale bar is equal to 25 μm.

To determine if injection with AdTGFβ1 resulted in overexpression of TGFβ1 in the lens, we performed immunohistochemistry on paraffin sections from naïve, AdTGFβ1 and AdDL treated eyes. Naïve lenses (Figure 3A) and those of AdDL (Figure 3B) did not show any TGFβ1 expression. However, animals treated with AdTGFβ1 showed positive expression of TGFβ1 in the lens plaques and adjacent lens epithelium at both days 4 (Figure 3C) and 21 (Figure 3D). Additionally, treatment with AdTGFβ1 resulted in strong expression of TGFβ1 protein in the corneal epithelium, stroma, and endothelium. Naïve and AdDL animals showed only faint expression limited to the cornified layer of the corneal epithelium and corneal endothelium and no expression in the stroma (data not shown).

**Figure 4:**
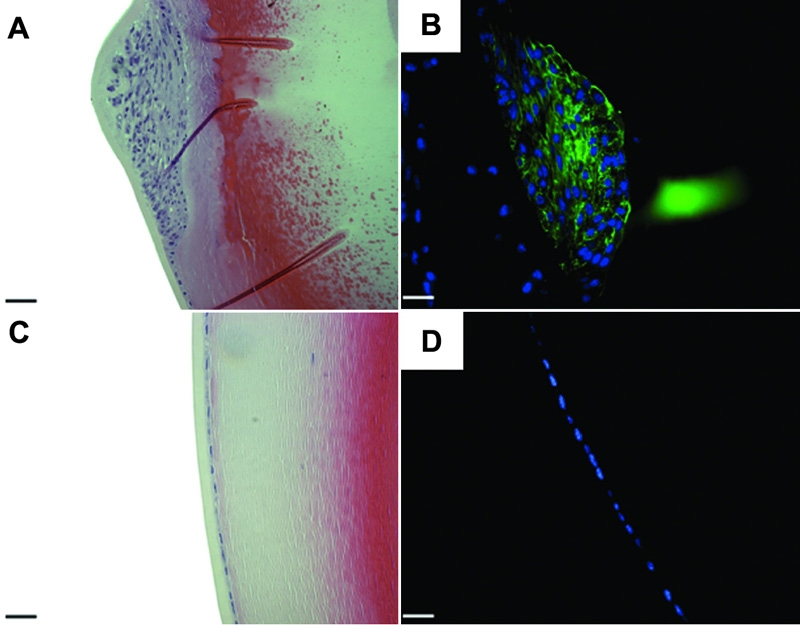
Effects of adenoviral gene transfer of active TGFβ1 after twenty one days. Histological sections from C57 mice injected with AdTGFβ1 (**A** and **B**) or AdDL (**C** and **D**). Sections were stained with H&E (**A** and **C**) or subjected to immunostaining for αSMA (**B** and **D**). At 21 days post injection AdTGFβ1 treated eyes demonstrate large plaques which express a considerable amount of αSMA in contrast to AdDL treated eyes. The green stain is αSMA and the blue stain is DAPI. The scale bar is equal to 25 μm.

To determine if the effects of AdTGFβ1 delivery to the anterior chamber were transient or chronic, we examined lenses of treated animals at 21 days post injection. Plaques seen in AdTGFβ1 treated eyes showed a marked increase in plaque size (Figure 4A). A proportion of these cells exhibited positive αSMA (Figure 4B) expression. AdDL treated eyes again showed no change in histological architecture (Figure 4C), lacked αSMA immunoreactivity (Figure 4D), and were strikingly similar to untreated lenses (data not shown).

### AdTGFβ1 induces expression of markers associated with fiber cell differentiation

**Figure 5:**
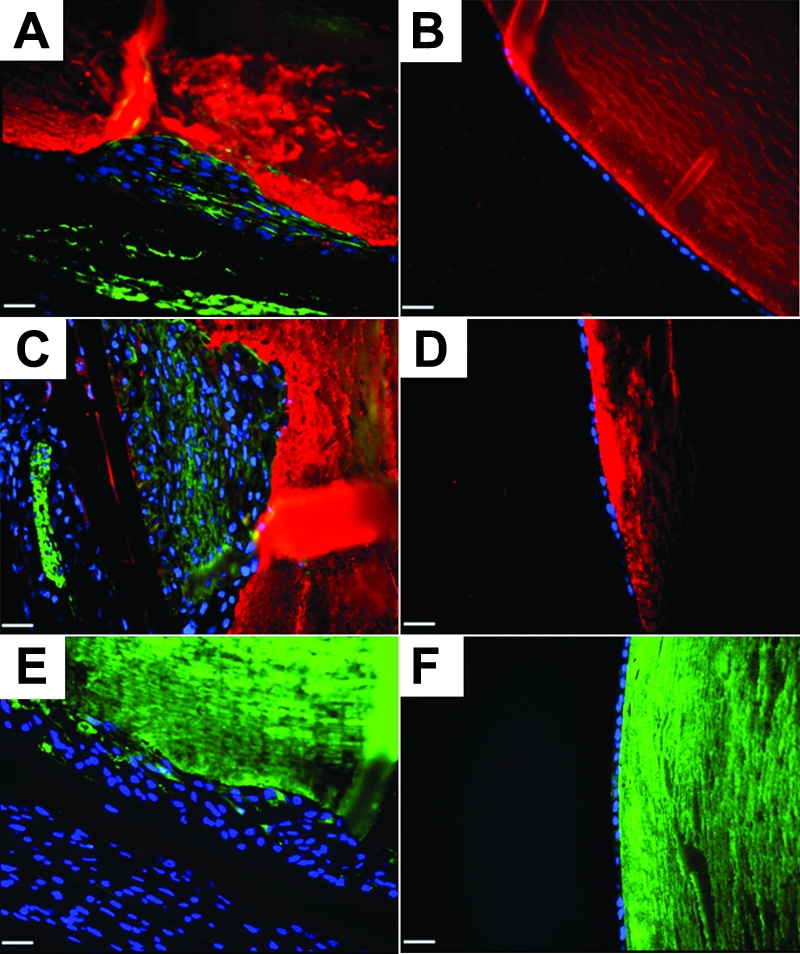
Crystallin expression in AdTGFβ1 treated eyes. Sections were stained with anti-β (**A**-**D**; red) or anti-γ-crystallin (**E** and **F**; green) antibodies and counterstained with DAPI (blue). Additionally, β-crystallin stained sections were colocalized with αSMA (**A**-**D**; green). Sections were taken at four days (**A** and **B**) and at twenty one days (**C**-**F**) of both AdTGFβ1 (**A**, **C**, and **E**) and AdDL (**B**, **D**, and **F**). Both β- and γ-crystallin expression can be seen with in the plaques of AdTGFβ1 but not AdDL treated eyes. Epithelia of AdDL treated eyes showed no presence of crystallin or αSMA expression. The scale bar is equal to 25 μm.

As shown above, not all of the cells in the plaques expressed αSMA. In order to clarify the nature of the cellular heterogeneity found in AdTGFβ1 induced ASC, we examined crystallin expression. It has been shown previously that LECs which do not express αSMA, expressed β-crystallin, an early fiber cell marker [31]. We therefore examined expression of β-crystallin in the subcapsular plaques of the AdTGFβ1 lenses at 4 and 21 days post injection. We observed detectable β-crystallin staining in a subpopulation of cells in the plaques of AdTGFβ1-treated eyes at both 4 (Figure 5A) and 21 days (Figure 5C) but no such detection in the epithelial monolayer of AdDL treated eyes at both time points (Figure 5B,D, respectively). Moreover, we were also able to detect expression of γ-crystallin in a subpopulation of cells in the plaques of AdTGFβ1 (Figure 5E) but not in the epithelial monolayer of AdDL (Figure 5F) lenses at 21 days.

### AdTGFβ1 induces aberrant expression of ECM components

**Figure 6:**
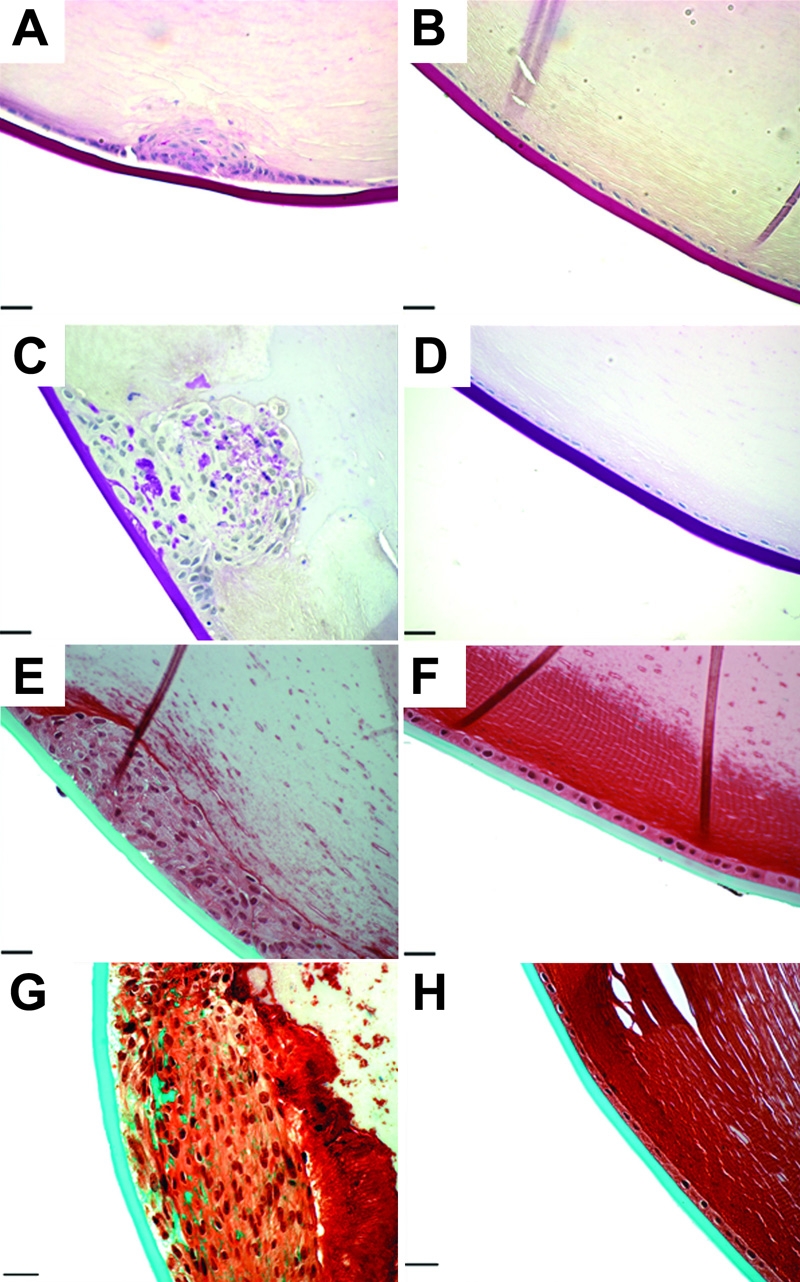
Matrix staining of AdTGFβ injected eyes. Sections of AdTGFβ1 (**A**, **C**, **E**, and **G**) and AdDL (**B**, **D**, **F**, and **H**) lenses taken on days 4 (**A**, **B**, **E**, and **F**) and 21 (**C**, **D**, **G**, and **H**) were stained with PAS (**A**-**D**) to detect carbohydrates (purple) and Masson's trichrome (**E**-**H**) to detect collagens (green). AdTGFβ1 treated lenses showed accumulation of matrix which was barely detectable on day 4, but prominent on day 21. In contrast, AdDL treated lenses showed no matrix accumulation at any time point. The scale bar is equal to 25 μm.

Inappropriate deposition of ECM is a characteristic feature of human ASC, and is also well documented in the transgenic mouse ASC model. We therefore examined the matrix composition of the ASC plaques in mice injected with AdTGFβ1 at both 4 and 21 days. To do so we employed the use of routine PAS histochemistry to detect carbohydrate deposition as well as Masson's trichrome to detect collagen deposition. Since both of these stains highlight the lens capsule in normal untreated lenses, we used this as an internal, positive control. At day 4 post-injection with AdTGFβ1 (Figure 6A,E) the subcapsular plaques demonstrated detectable amounts of matrix deposition in contrast to treatment with AdDL (Figure 6B,F). However, at 21 days post injection, the ASC plaques in the AdTGFβ1-treated eyes exhibited notable deposits of both carbohydrate (Figure 6C) and collagen (Figure 6G). AdDL treated animals showed no matrix accumulation at this time (Figure 6D,H).

**Figure 7:**
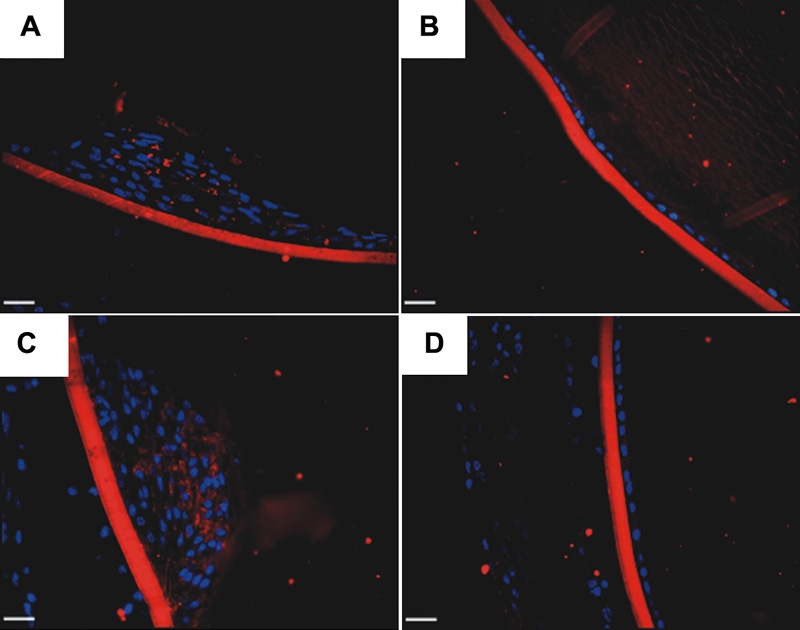
Collagen IV expression. Immunolocalization of collagen IV was performed on paraffin sections of AdTGFβ1 (**A** and **C**) and AdDL (**B** and **D**) lenses on days 4 (**A** and **B**) and 21 (**C** and **D**). AdTGFβ1 treated eyes showed a marked accumulation of collagen IV in the plaques which was absent in epithelia of AdDL treated eyes. The red staining is collagen IV and the blue staining is DAPI. The scale bar is equal to 25 μm.

Collagen IV is the most abundant collagen found in the lens capsule but is absent in the lens epithelium. We therefore used immunohistochemistry to reveal whether collagen type IV was aberrantly expressed in the epithelial cells comprising the plaque. Animals treated with AdTGFβ1 showed collagen IV accumulation at both days 4 and 21 (Figure 7A,C, respectively) whereas those animals treated with AdDL showed no accumulation at either time point (Figure 7B,D).

### AdTGFβ1 induces ASC in Smad3-knockout mice

**Figure 8:**
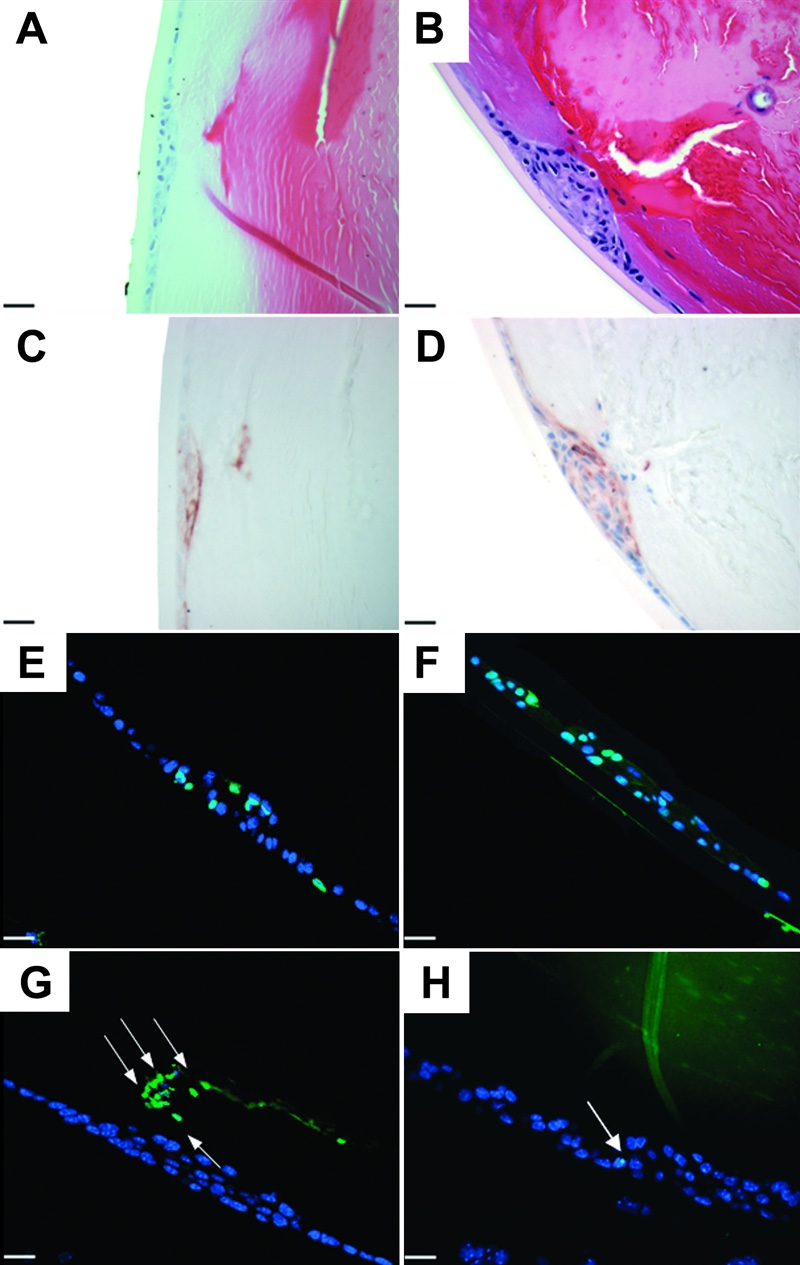
Effects of adenoviral gene transfer of active TGFβ1 to Smad3KO eyes after four days. Histological sections from Smad3KO (**A**, **C**, **E**, and **G**) and WT (**B**, **D**, **F**, and **H**) treated with AdTGFβ1. Both groups developed αSMA (brown; **C** and **D**) expressing plaques, and showed no difference in cellular proliferation by the PCNA (green) stain (**E** and **F**). Smad3 KO (**G**) animals demonstrated more TUNEL (green) positive cells (arrows) compared to WT (**H**). The scale bar is equal to 25 μm.

With our findings that AdTGFβ1 delivery to the anterior chamber resulted in the development of ASC reminiscent of that observed in other animal models and human ASC, we next employed this model to further confirm our earlier findings [23] that ASC can develop in the absence of the Smad3 signaling intermediate. We tested this by delivering AdTGFβ1 to the anterior chamber of Smad3KO eyes. Four days post injection of AdTGFβ1, the lenses of Smad3KO and WT mice showed distinct subcapsular plaques consisting of a focal multi-layering of cells in the central, anterior region (Figure 8A,B, respectively) that were positive for αSMA expression (Figure 8C,D, respectively). However, Smad3KO mice exhibited plaques that were reduced in size relative to those seen in WT littermates. To further investigate why the plaques in the Smad3KO mice were smaller than WT littermates we performed experiments to assess both cellular proliferation and apoptosis. To detect cellular proliferation, we immunostained sections with proliferating cell nuclear antigen (PCNA). These experiments showed no observable difference in the amount of cells immunoreactive to PCNA in the lens plaques of AdTGFβ1-treated Smad3KO mice (Figure 8E) versus plaques in the AdTGFβ1-treated WT littermates (Figure 8F). However, TUNEL staining, used to detect apoptotic cells, revealed that plaques from the AdTGFβ1-treated Smad3KO mice had substantially more apoptotic cells than plaques of the AdTGFβ1-treated wild-type littermates (Figure 8G,H, respectively).

## Discussion

In this study we have developed a novel model for ASC. Adenoviral delivery of TGFβ1 to the anterior chamber of the mouse eye produced the formation of distinct subcapsular cataracts, 4 days post injection, which exhibited features of ASC plaques described in humans and other rodent models, including an induction in αSMA expression and aberrant deposition of ECM. Unlike TGFβ1-transgenic mouse models, whereby active TGFβ1 is overexpressed in lens fiber cells during embryogenesis [19,20], the AdTGFβ1 gene delivery model does not require the postnatal period (21 days after birth) that the transgenic models require for subcapsular plaques to be observed. Also, since heterozygote lines of the transgenic mice are typically used for examination, due to the severity of the homozygous phenotype, each mouse or embryo of the TGFβ1-transgenic line to be examined must be genotyped. The AdTGFβ1 model does not require crossbreeding or genotyping and can therefore be more easily employed in studies utilizing genetically modified mice.

Intravitreal injection of recombinant TGFβ into the rat eye [32] has been shown to produce ASC following 15 weeks post injection, however the ASC were not well defined and were accompanied by a number of other lens defects, including cortical clouding, disrupted bow region, nucleated fiber cells, vacuolation of fiber cells, and posterior subcapsular cataracts containing swollen and degenerating cells. These features are not typically found in patients with ASC and none of these features were observed in our AdTGFβ model, even at 3 weeks post injection. Injection into the vitreous cavity as opposed to the anterior chamber may have different outcomes. For example, Oksala et al. [33] found that intracameral injection of calcitonin gene-related peptide (CGRP) increased, whereas intravitreal injection of CGRP decreased intraocular pressure. Thus, the difference in response to TGFβ in the two models may be related to the site of delivery. Intracameral delivery of recombinant TGFβ in rodents has not been described, and this may be due to the fact that it is quickly turned over in the anterior chamber through drainage of the aqueous humor [34].

Another in vivo model of ASC is a lens injury model in mice [35]. In this model, a hypodermic needle is inserted trans-corneally to disrupt the lens capsule, resulting in the EMT of LECs and ASC formation. While this model produces many of the features observed in human ASC, one aspect of this model is rupture of the lens capsule, which is not associated with ASC development. Disruption of the capsule in the lens injury model results in exposure of the lens crystallins to resident antigen presenting cells and may elicit an immune response that may not normally be found in ASC patients [36,37]. Similarly, the recent ASC model developed using topical alkali application to the ocular surface elicits wound responses in multiple tissues of the anterior segment [22]. Use of adenoviral vectors in vivo [38] typically induces both innate and adaptive immune responses to the viral proteins [39]. Anterior chamber injections, however, typically do not produce systemic responses and do not induce delayed type hypersensitivity when animals are re-challenged with antigen [40]. Thus, in the AdTGFβ1 model, the lens capsule remains intact and only a minimal immune response directed against the adenoviral vector is observed. Additionally, since transgene expression is transient, a small initial dose may be all that is required [41].

While many laboratories have shown that adenovirus can transduce lens epithelial cells in culture [42], the ability of the adenovirus to transduce the lens epithelium in vivo has been a matter of debate [43]. However, using both AdGFP and AdLacZ vectors we demonstrated that adenoviral delivery to the anterior chamber resulted in transduction of the mouse lens epithelium, in the absence of a capsular break. Additionally, AdTGFβ1 treated eyes showed upregulated levels of TGFβ1 in the lens epithelium. Recent work has shown that intravitreal injection of AAV vectors encoding enhanced green fluorescent protein can also transduce the lens epithelium [44]. In addition to the lens, we found that multiple structures of the anterior chamber, including the corneal endothelium and stromal cells, were also transduced by AdLacZ. Thus, delivery of the AdTGFβ1 may have resulted in the transduction of many of these tissues which as a result may have contributed to development of the AdTGFβ1-induced ASC. At the present time it is not known whether transduction of the lens is required for development of ASC. Nonetheless, the intracameral delivery of AdTGFβ1 represents a quick and reproducible model for ASC in mice.

Employment of the AdTGFβ1 model to the Smad3 deficient mice confirmed our earlier findings demonstrating that while Smad3 is sufficient for, it is not necessary for the EMT of LECs during ASC formation in vivo. In our recent study, TGFβ1 transgenic mice expressing active TGFβ1 under the αA-crystallin promoter, were bred onto a Smad3KO background and exhibited ASC plaques, albeit they were smaller than those found in their wild-type littermates [23]. This was correlated with an increase in apoptosis in the subcapsular plaques and a decrease in expression of collagen type IV. Similar to these findings, we also observe that AdTGFβ1 induced ASC were smaller in Smad3KO animals compared to their wildtype littermates and exhibited higher numbers of apoptotic cells. We further extended this finding to show that proliferation of cells in the ASC, as assessed by PCNA immunostaining, did not appear to differ between the AdTGFβ1-treated Smad3KO and wild-type mice. Thus together these data suggest that decreased cell survival is likely to be the underlying cause of the smaller sized plaques in the Smad3KO mice.

Findings for the models described above, as well as the recently developed alkali burn model, are in contrast to earlier work by Saika and colleagues which showed that Smad3 deficient mice were resistant to ASC formation in response to lens injury [45]. One possible explanation for the difference, as also pointed out by these authors [22], is that the levels of active TGFβ produced in each of the models may be different. The adenoviral gene transfer and transgenic approaches may have produced larger amounts of active TGFβ than that of the lens injury model. Indeed different transgenic promoters with different strengths were shown to result in variability in the severity of anterior chamber and lens defects in mice [19,20]. Finally, individual susceptibility among mouse strains has been well documented and may explain some of these differences. For example, strains that are susceptible to fibrosis-inducing stimuli include C57BL/6, which were used in these studies [46-50].

In summary, we have developed a novel model of ASC involving adenoviral delivery of active porcine TGFβ1 to the anterior segment of rodent eye. AdTGFβ1 induced ASC consisting of epithelial multilayering beneath the lens capsule and induction of αSMA expression within 4 days, which was sufficient to further induce aberrant deposition of ECM in mice out to 21 days post infection. We also confirm that TGFβ1-induced EMT of LECs as evidenced by αSMA expression is not Smad3-dependent in that Smad3 deficient mice can develop small ASC plaques that are αSMA positive, further demonstrating that additional TGFβ signaling cascades participate in the EMT of LECs and ASC formation.

## References

[1] Derynck R (1994). TGF-beta-receptor-mediated signaling.. Trends Biochem Sci.

[2] Leask A, Abraham DJ (2004). TGF-beta signaling and the fibrotic response.. FASEB J.

[3] Saika S (2006). TGFbeta pathobiology in the eye.. Lab Invest.

[4] Saika S, Yamanaka O, Ikeda K, Kim-Mitsuyama S, Flanders KC, Yoo J, Roberts AB, Nishikawa-Ishida I, Ohnishi Y, Muragaki Y, Ooshima A (2005). Inhibition of p38MAP kinase suppresses fibrotic reaction of retinal pigment epithelial cells.. Lab Invest.

[5] Priglinger SG, Alge CS, Kreutzer TC, Neubauer AS, Haritoglou C, Kampik A, Welge-Luessen U (2006). Keratinocyte transglutaminase in proliferative vitreoretinopathy.. Invest Ophthalmol Vis Sci.

[6] Lutjen-Drecoll E (2005). Morphological changes in glaucomatous eyes and the role of TGFbeta2 for the pathogenesis of the disease.. Exp Eye Res.

[7] Wallentin N, Wickstrom K, Lundberg C (1998). Effect of cataract surgery on aqueous TGF-beta and lens epithelial cell proliferation.. Invest Ophthalmol Vis Sci.

[8] Cousins SW, McCabe MM, Danielpour D, Streilein JW (1991). Identification of transforming growth factor-beta as an immunosuppressive factor in aqueous humor.. Invest Ophthalmol Vis Sci.

[9] Jampel HD, Roche N, Stark WJ, Roberts AB (1990). Transforming growth factor-beta in human aqueous humor.. Curr Eye Res.

[10] Thompson JT, Glaser BM, Sjaarda RN, Murphy RP (1995). Progression of nuclear sclerosis and long-term visual results of vitrectomy with transforming growth factor beta-2 for macular holes.. Am J Ophthalmol.

[11] de Iongh RU, Wederell E, Lovicu FJ, McAvoy JW (2005). Transforming growth factor-beta-induced epithelial-mesenchymal transition in the lens: a model for cataract formation.. Cells Tissues Organs.

[12] Sacu S, Menapace R, Findl O, Georgopoulos M, Buehl W, Kriechbaum K, Rainer G (2004). Influence of optic edge design and anterior capsule polishing on posterior capsule fibrosis.. J Cataract Refract Surg.

[13] Kappelhof JP, Vrensen GF (1992). The pathology of after-cataract. A minireview.. Acta Ophthalmol Suppl.

[14] Liu J, Hales AM, Chamberlain CG, McAvoy JW (1994). Induction of cataract-like changes in rat lens epithelial explants by transforming growth factor beta.. Invest Ophthalmol Vis Sci.

[15] Hales AM, Chamberlain CG, McAvoy JW (1995). Cataract induction in lenses cultured with transforming growth factor-beta.. Invest Ophthalmol Vis Sci.

[16] Gordon-Thomson C, de Iongh RU, Hales AM, Chamberlain CG, McAvoy JW (1998). Differential cataractogenic potency of TGF-beta1, -beta2, and -beta3 and their expression in the postnatal rat eye.. Invest Ophthalmol Vis Sci.

[17] Saika S, Okada Y, Miyamoto T, Ohnishi Y, Ooshima A, McAvoy JW (2001). Smad translocation and growth suppression in lens epithelial cells by endogenous TGFbeta2 during wound repair.. Exp Eye Res.

[18] Dwivedi DJ, Pino G, Banh A, Nathu Z, Howchin D, Margetts P, Sivak JG, West-Mays JA (2006). Matrix metalloproteinase inhibitors suppress transforming growth factor-beta-induced subcapsular cataract formation.. Am J Pathol.

[19] Srinivasan Y, Lovicu FJ, Overbeek PA (1998). Lens-specific expression of transforming growth factor beta1 in transgenic mice causes anterior subcapsular cataracts.. J Clin Invest.

[20] Flugel-Koch C, Ohlmann A, Piatigorsky J, Tamm ER (2002). Disruption of anterior segment development by TGF-beta1 overexpression in the eyes of transgenic mice.. Dev Dyn.

[21] Saika S, Ikeda K, Yamanaka O, Sato M, Muragaki Y, Ohnishi Y, Ooshima A, Nakajima Y, Namikawa K, Kiyama H, Flanders KC, Roberts AB (2004). Transient adenoviral gene transfer of Smad7 prevents injury-induced epithelial-mesenchymal transition of lens epithelium in mice.. Lab Invest.

[22] Shirai K, Saika S, Tanaka T, Okada Y, Flanders KC, Ooshima A, Ohnishi Y (2006). A new model of anterior subcapsular cataract: involvement of TGFbeta/Smad signaling.. Mol Vis.

[23] Banh A, Deschamps PA, Gauldie J, Overbeek PA, Sivak JG, West-Mays JA (2006). Lens-specific expression of TGF-beta induces anterior subcapsular cataract formation in the absence of Smad3.. Invest Ophthalmol Vis Sci.

[24] Sime PJ, Xing Z, Graham FL, Csaky KG, Gauldie J (1997). Adenovector-mediated gene transfer of active transforming growth factor-beta1 induces prolonged severe fibrosis in rat lung.. J Clin Invest.

[25] Margetts PJ, Kolb M, Galt T, Hoff CM, Shockley TR, Gauldie J (2001). Gene transfer of transforming growth factor-beta1 to the rat peritoneum: effects on membrane function.. J Am Soc Nephrol.

[26] Bonniaud P, Margetts PJ, Kolb M, Haberberger T, Kelly M, Robertson J, Gauldie J (2003). Adenoviral gene transfer of connective tissue growth factor in the lung induces transient fibrosis.. Am J Respir Crit Care Med.

[27] Kolb M, Margetts PJ, Sime PJ, Gauldie J (2001). Proteoglycans decorin and biglycan differentially modulate TGF-beta-mediated fibrotic responses in the lung.. Am J Physiol Lung Cell Mol Physiol.

[28] Brunner AM, Marquardt H, Malacko AR, Lioubin MN, Purchio AF (1989). Site-directed mutagenesis of cysteine residues in the pro region of the transforming growth factor beta 1 precursor. Expression and characterization of mutant proteins.. J Biol Chem.

[29] Bett AJ, Haddara W, Prevec L, Graham FL (1994). An efficient and flexible system for construction of adenovirus vectors with insertions or deletions in early regions 1 and 3.. Proc Natl Acad Sci USA.

[30] Yang X, Letterio JJ, Lechleider RJ, Chen L, Hayman R, Gu H, Roberts AB, Deng C (1999). Targeted disruption of SMAD3 results in impaired mucosal immunity and diminished T cell responsiveness to TGF-beta.. EMBO J.

[31] Lovicu FJ, Steven P, Saika S, McAvoy JW (2004). Aberrant lens fiber differentiation in anterior subcapsular cataract formation: a process dependent on reduced levels of Pax6.. Invest Ophthalmol Vis Sci.

[32] Hales AM, Chamberlain CG, Dreher B, McAvoy JW (1999). Intravitreal injection of TGFbeta induces cataract in rats.. Invest Ophthalmol Vis Sci.

[33] Oksala O, Heino P, Uusitalo H, Palkama A (1998). Effect of intracameral and intravitreal injection of calcitonin gene-related peptide on the intraocular pressure and outflow facility of aqueous humor in the rabbit.. Exp Eye Res.

[34] Aihara M, Lindsey JD, Weinreb RN (2003). Aqueous humor dynamics in mice.. Invest Ophthalmol Vis Sci.

[35] Rafferty NS, Goossens W (1975). Ultrastructural studies of traumatic cataractogenesis: observations of a repair process in mouse lens.. Am J Anat.

[36] Goldschmidt L, Goldbaum M, Walker SM, Weigle WO (1982). The immune response to homologous lens crystallin. I. Antibody production after lens injury.. J Immunol.

[37] Goldschmidt L, Goldbaum M, Walker SM, Weigle WO (1982). The immune response to homologous lens crystallin. II. A model of ocular inflammation involving eye injuries at separate times.. J Immunol.

[38] Bennett J (2003). Immune response following intraocular delivery of recombinant viral vectors.. Gene Ther.

[39] Ritter T, Lehmann M, Volk HD (2002). Improvements in gene therapy: averting the immune response to adenoviral vectors.. BioDrugs.

[40] Suber ML, Hurwitz MY, Chevez-Barrios P, Hurwitz RL (2001). Immune consequences of intraocular administration of modified adenoviral vectors.. Hum Gene Ther.

[41] Wormstone IM, Anderson IK, Eldred JA, Dawes LJ, Duncan G (2006). Short-term exposure to transforming growth factor beta induces long-term fibrotic responses.. Exp Eye Res.

[42] Carrington LM, Southgate T, Saxby LA, Abul-Hassan K, Maleniak TC, Castro MG, Boulton ME (2000). Adenovirus-mediated gene transfer to human lens epithelial cells in organ culture.. J Cataract Refract Surg.

[43] Borras T, Tamm ER, Zigler JS (1996). Ocular adenovirus gene transfer varies in efficiency and inflammatory response.. Invest Ophthalmol Vis Sci.

[44] Auricchio A, Kobinger G, Anand V, Hildinger M, O'Connor E, Maguire AM, Wilson JM, Bennett J (2001). Exchange of surface proteins impacts on viral vector cellular specificity and transduction characteristics: the retina as a model.. Hum Mol Genet.

[45] Saika S, Kono-Saika S, Ohnishi Y, Sato M, Muragaki Y, Ooshima A, Flanders KC, Yoo J, Anzano M, Liu CY, Kao WW, Roberts AB (2004). Smad3 signaling is required for epithelial-mesenchymal transition of lens epithelium after injury.. Am J Pathol.

[46] Schrier DJ, Kunkel RG, Phan SH (1983). The role of strain variation in murine bleomycin-induced pulmonary fibrosis.. Am Rev Respir Dis.

[47] Bonniaud P, Martin G, Margetts PJ, Ask K, Robertson J, Gauldie J, Kolb M (2004). Connective tissue growth factor is crucial to inducing a profibrotic environment in "fibrosis-resistant" BALB/c mouse lungs.. Am J Respir Cell Mol Biol.

[48] Kolb M, Bonniaud P, Galt T, Sime PJ, Kelly MM, Margetts PJ, Gauldie J (2002). Differences in the fibrogenic response after transfer of active transforming growth factor-beta1 gene to lungs of "fibrosis-prone" and "fibrosis-resistant" mouse strains.. Am J Respir Cell Mol Biol.

[49] Daly HE, Baecher-Allan CM, Barth RK, D'Angio CT, Finkelstein JN (1997). Bleomycin induces strain-dependent alterations in the pattern of epithelial cell-specific marker expression in mouse lung.. Toxicol Appl Pharmacol.

[50] Warshamana GS, Pociask DA, Sime P, Schwartz DA, Brody AR (2002). Susceptibility to asbestos-induced and transforming growth factor-beta1-induced fibroproliferative lung disease in two strains of mice.. Am J Respir Cell Mol Biol.

